# Biomechanical analyses point to dense collagen fibre architecture in an Ediacaran frond

**DOI:** 10.1098/rspb.2026.1630

**Published:** 2026-10-14

**Authors:** Zekun Wang, Princess Buma-at, Nile Stephenson, Frances S. Dunn, Qianze Zhuang, Emily G. Mitchell, Imran A. Rahman

**Affiliations:** ^1^ The Natural History Museum, London SW7 5BD, UK; ^2^ Department of Zoology and University Museum of Zoology, University of Cambridge, Cambridge CB2 3EJ, UK; ^3^ King's College, University of Cambridge, Cambridge, CB2 1ST, UK; ^4^ Oxford University Museum of Natural History, University of Oxford, Oxford OX1 3PW, UK; ^5^ State Key Laboratory of Coastal and Offshore Engineering, Dalian University of Technology, Dalian 116024, People's Republic of China

**Keywords:** Ediacaran, frondose organism, collagen, computational fluid dynamics, dimensional analysis

## Abstract

Ediacaran macrofossils record the earliest large and complex organisms on Earth. Despite extensive study, fundamental aspects of their internal anatomy and tissue composition remain poorly known, limiting understanding of how these organisms were constructed and functioned, as well as how their complex anatomies evolved. Here, we establish a new quantitative biomechanical framework to infer tissue properties in the Ediacaran frond *Charniodiscus*. Analyses of fossil morphology, numerical simulations and dimensional analysis indicate that the commonly preserved twisted forms reflect three-dimensional torsional deformation driven by turbidity flow-induced instability. By mapping deformation modes in dimensionless phase space, we calculate a Young's modulus consistent with densely aligned collagen fibres, under a eumetazoan framework. These results point to the presence of a load-bearing stem and substantial functional specialization. The inferred mechanical properties are largely incompatible with non-animal, sponge or placozoan tissues, and independently strengthen the proposal that *Charniodiscus* was a total-group eumetazoan. The presence of these tissues marked a foundational evolutionary step, providing the scaffolding for increased size, complexity and innovation in early animals.

## 1. Introduction

The Avalon assemblage (approx. 574–560 Ma) consists of the oldest known deep-water animal communities, which are found mainly in Newfoundland (Canada) and Charnwood Forest (UK) [1–4]. While the biological affinities of the constituent organisms have historically proved highly controversial because of the presence of unusual or unique morphological characters such as body plan-scale glide symmetry [5–14], there is growing morphological, developmental and phylogenetic evidence supporting the interpretation of at least some Ediacaran taxa as eumetazoans [13,15–18]. However, very little is known about the tissue organization and properties of Ediacaran organisms because internal anatomical structures are rarely preserved [1,13,16,19]. Consequently, insights into their anatomical compartmentalization are obtained primarily from morphological features, such as the fascicled branches connecting individual ‘peas’ in the ‘pea-pod’ branches in *Arborea*, and the fluidized sediment emanating from a break in its holdfast disc [15]. While such morphological evidence may offer an independent test of organismal affinities—as in developmental analyses of *Arborea* and *Charnia* [15,16]—the general scarcity of preserved internal structures in these Ediacaran fronds still hampers our understanding of body-tissue evolution in early animals, the underlying cell types and developmental programs, and their value as phylogenetically informative traits.

The majority of Avalonian taxa are characterized by a frondose body plan [20]. *Charniodiscus* species are common on bedding planes, with broad geographic and stratigraphic ranges through the late Ediacaran [2]. They exhibit a bilobate frond and a straight, erect stem. Across multiple different localities in Mistaken Point, Canada, *Charniodiscus* specimens are preserved as bent or twisted forms (figure 1). Fronds from this locality are inferred to have lived in contour currents [21,22], with the observed deformations occurring as the organisms were knocked over by the turbidity flows thought to have killed them [21–23]. Therefore, Mistaken Point *Charniodiscus* provide an ideal model for investigating the material properties of the Ediacaran frondose organism. By combining numerical simulations with dimensional analysis, we assess how flow conditions, organismal size and mechanical properties governed fluid–structure interactions in *Charniodiscus*, allowing us to identify key dimensionless numbers that can be used to classify different deformation modes. The torsional geometries observed in Mistaken Point specimens are then used to constrain the material properties of *Charniodiscus*, and the resulting range of Young's modulus values is compared with those of extant eukaryotes within a biomechanical phase space. As tissue material properties are emergent from biomaterial type, tissue structure and developmental mechanisms, which are themselves evolutionarily conserved, the results allow us to rigorously—and independently—constrain the phylogenetic affinities of *Charniodiscus*, with important implications for uncovering the evolution of key animal characters.

**Figure 1.**
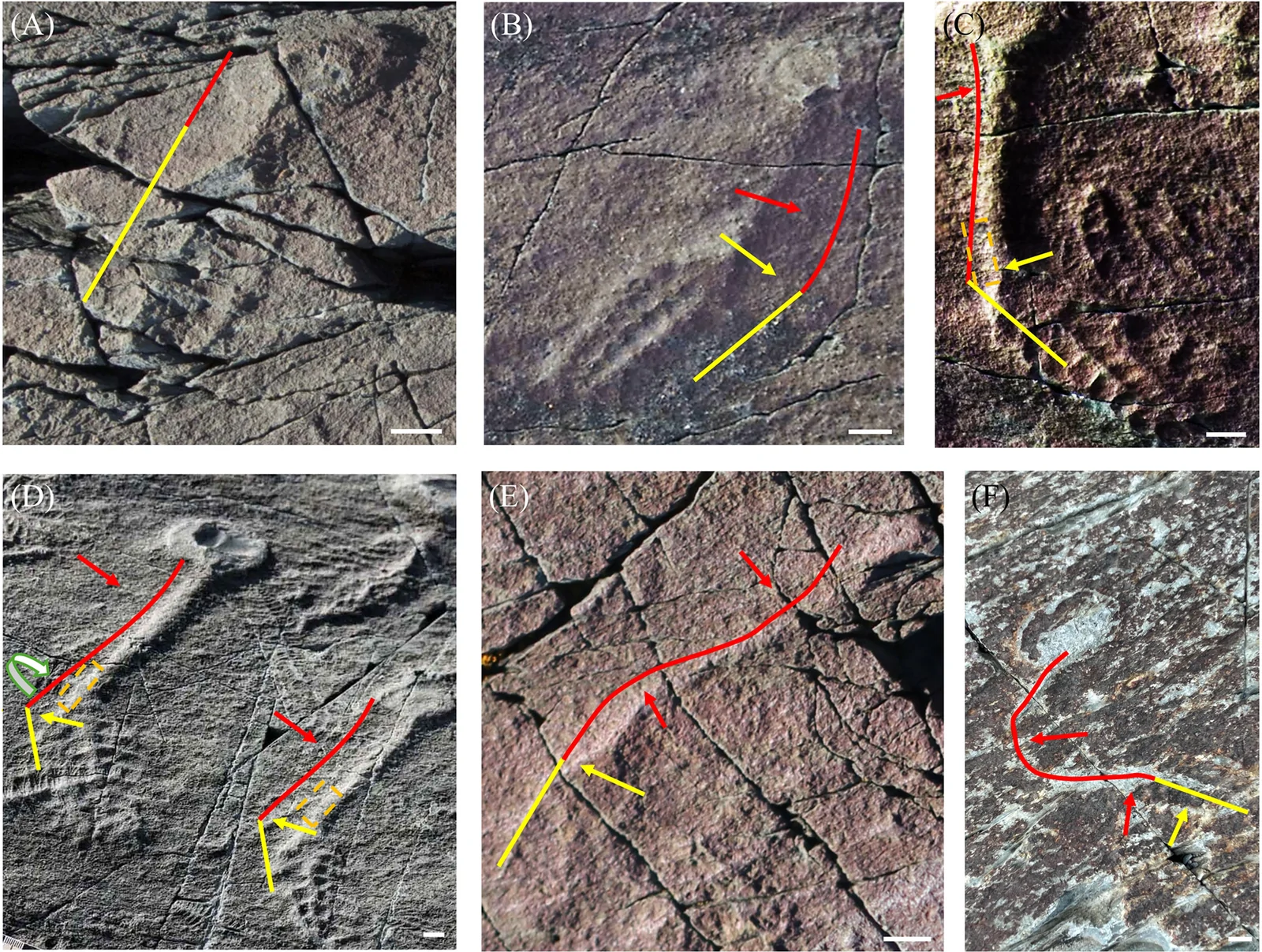
Preserved forms of *Charniodiscus* from the Mistaken Point E (A–E) and Mistaken Point North 2 (F) surfaces. (A) Straight and undeformed *C*. *spinosus*. (B–E) *C. procerus* that underwent bending (B), torsion (C, D) or secondary torsion (E, F). (F) *C*. *procerus* under secondary torsion with two opposite curvatures along the stem. The red and yellow arrows indicate the curvature of the central line of the stem and the stem–frond junction, respectively. The orange dashed boxes indicate the location of a small buckling at the kinked stem–frond junction. Scale bars: 1 cm.

## 2. Materials and methods

### (a) Materials

We analysed 112 specimens of *Charniodiscus procerus* and *Charniodiscus spinosus* from the Mistaken Point E surface from Newfoundland, Canada. We also examined severely deformed specimens from the Mistaken Point North 2 surface. These surfaces were mapped following [24], using a LiDAR Faro Focus 330x, creating three-dimensional surface meshes that captured the entire surfaces accurately to approximately 1 mm. For the Mistaken Point E surface, high-resolution scans were also acquired using a Faro Scan Arm Laser Line Probe in approximately 1 × 1 m^2^ grids, incorporating control points common to the LiDAR scan to enable accurate alignment of the two data sets, and this resulted in a surface mesh with a mean resolution of 0.050 mm. The identity, position, orientation and dimensions (disc width, disc length, stem length, stem width, frond length and frond width) of fossils were determined in Inkscape v. 0.92.3 using a two-dimensional projection of the combined data set, resulting in a two-dimensional vector map that was used for our subsequent analyses. In addition, we recorded the dimensions of two specimens from the Mistaken Point North 2 surface in order to fully characterize the preserved variation exhibited by this genus.

Mechanical properties of modern tissues were collected from references [25–44] and are presented in table S2 in the supplementary material.

### (b) Numerical simulations

We used the well-validated codes in Solids4Foam v. 2.0 [45] based on the foam-extend-v4.0 framework to simulate fluid–frond interactions to test how flow conditions, frond size and material properties influenced the mode of deformation. The finite volume method was used to solve the fluid motion and frond deformation, integrated through an arbitrary Lagrangian–Eulerian framework [46]. The *Charniodiscus procerus* model was created in Blender v. 3.5 based on the geometry of the left-hand frond in figure 1D, which was also used to specify the model dimensions (i.e. 25 cm tall). The fluid domain was 30 cm high and 60 cm wide. The mesh was generated using the snappyHexMesh tool in foam-extend-v4.0. The same material properties were assigned to the whole model. To accurately replicate the vertical flow profile, the inlet flow was set 30 cm upstream of the *Charniodiscus* model according to boundary layer theory [47], with flow transitioning smoothly from a linear profile to a logarithmic region characterized by the dimensionless parameter *y*^+^ ( = *ρzU_τ_*/*µ*), where *ρ* = 1027.5 kg m^–3^ and *µ* = 0.001594 kg m^–1^ s^–1^ are the density and viscosity of sea water, respectively, estimated based on present-day high-latitude bottom waters [48–50], *z* is the distance from the substrate, and *U_τ_* is the frictional velocity at the sediment–fluid interface. Simulations were run for a duration of 100 seconds, although the deformation reached a steady state before this time, particularly under low-flow conditions or when the kinked frond became self-locked by the substrate. The mean grid resolution was 500 µm, which provided a good fit to the analytical results used for verification (supplementary material, figure S1), and the time step was chosen to maintain a Courant number (the ratio of the product of the maximum velocity and the time step to the minimum grid size) below 0.015. The solid domain was modelled using a total Lagrangian finite-strain formulation, and as the viscous effects are expected to be limited for stems (three-dimensional preservation indicates limited water contents in these tissues), the neo‑Hookean model and a segregated solution strategy (*nonLinGeomTotalLagSolid*) were used. The fluid flow was solved using the PIMPLE algorithm for pressure–velocity coupling, and smoothed solvers (e.g. Gauss–Seidel smoother) were employed for the computation of fluid-related variables. A laminar or *k*-*ω* SST model was used depending on the Reynolds number. Simulation results were visualized using ParaView v. 5.8 [51]. Model set-ups and data are available at Zenodo [52].

### (c) Analytical solutions for bending

The Euler–Bernoulli equation [53] ensures a mathematically smooth profile for the bent fronds:

(2.1) $$\frac{d^{2}w}{dx^{2}}=\frac{1}{EI}\iint qdx,$$

where deflection *w* is the second-order integral of a function along the axis (*x* is the distance) involving the Young's modulus *E*, second axial moment of area *I* and the distributed drag from the current *q*. For a predominantly solid stem, $I=\frac{\pi }{64}(d^{4}-d_{i}^{4})$, where *d* and *d_i_* are the diameter of the stem and inner cavity. The inner cavity has a relatively small impact; for example, if *d*_i_ = 0.2*d*, the relative difference from a solid stem is only 0.16%. Therefore, abrupt changes in material properties, shapes or fluid conditions will still result in a solution with at least one-order smoothness (i.e. continuous one-order derivatives, meaning when two points along the curve are close enough, their tangents eventually overlap). This smoothness ensures that no matter how flow conditions or frond parameters vary, the deflection curve (deformation profile) along the central axis will always have no abrupt transitions (‘kinks’) where no derivative exists. On the other hand, if the frond is flipped or folded, the curvature would also flip accordingly. At the point when the frond is flipped (‘kink’), a derivative does not exist (the tangent lines of the two points before and after the non-differentiable point will never overlap, no matter how close the two points are). Therefore, smoothness or the existence of a non-differentiable point differs from pure bending and one-order torsion. For verification, we used the analytical solution for a uniformly loaded solid circular beam (supplementary material, figure S1B) according to equation (2.1).

The Euler–Bernoulli equation ensures a unique solution, establishing a one-to-one correspondence between the frond deformation, flexural rigidity, boundary conditions (the holdfast) and the applied loads (both their direction and magnitude) [53]. This fixed relationship provides the theoretical foundation for our analysis.

### (d) Dimensional analysis

We used the Buckingham Π theorem [54,55] to derive the dimensionless numbers governing fluid–frond interactions. With the obtained dimensionless numbers, we can establish a phase map to classify different deformation modes and predict the material properties of the frond under the expected flow conditions. To perform dimensional analysis, we identified eight factors thought to influence the bending or torsion of the frond: (i) the angle between the combined contour current–turbidity flow that led to their death and the normal vector of the frond (parallel to contour currents), *θ*; (ii) the fluid velocity, *U*; (iii) the fluid density, *ρ*; (iv) the fluid viscosity, *µ*; (v) the stem length, *l*; (vi) stem diameter, *d*; (vii) the surface area of the frond, *A*; and (viii) the shear modulus of the frond, *G* = 0.5*E*/(1 + *υ*), where *E* is the elastic (Young's) modulus and *υ* is the Poisson's ratio, which is assumed to be 0.4, consistent with the typical range for most natural materials (0.3 to 0.5) [56]. Except for *θ*, which is already dimensionless, the remaining seven parameters have three base units (*kg*, *m* and *s*), resulting in four additional dimensionless numbers that govern the frond–fluid interactions.

Let the dimensionless number $\Pi =U^{x_{1}}\mu^{x_{2}}d^{x_{3}}\rho^{x_{4}}l^{x_{5}}A^{x_{6}}G^{x_{7}}$, and write it in a matrix equation:

(2.2) $$\begin{array}{ccc} & s: & \\ & kg: & \\ & m: & \end{array}\left[\begin{array}{ccccccccc} & -1 & -1 & 0 & 0 & 0 & 0 & -2 & \\ & 0 & 1 & 0 & 1 & 0 & 0 & 1 & \\ & 1 & -1 & 1 & -3 & 1 & 2 & -1 & \end{array}\right]\left[\begin{array}{ccc} & x_{1} & \\ & x_{2} & \\ & x_{3} & \\ & x_{4} & \\ & x_{5} & \\ & x_{6} & \\ & x_{7} & \end{array}\right]=\left[\begin{array}{ccc} & 0 & \\ & 0 & \\ & 0 & \end{array}\right]\to \left[\begin{array}{ccccccccc} & 1 & 0 & 0 & -1 & 0 & 0 & 1 & \\ & 0 & 1 & 0 & 1 & 0 & 0 & 1 & \\ & 0 & 0 & 1 & -1 & 1 & 2 & -1 & \end{array}\right]\left[\begin{array}{ccc} & x_{1} & \\ & x_{2} & \\ & x_{3} & \\ & x_{4} & \\ & x_{5} & \\ & x_{6} & \\ & x_{7} & \end{array}\right]=\left[\begin{array}{ccc} & 0 & \\ & 0 & \\ & 0 & \end{array}\right].$$

Thus, we have four fundamental sets of solutions for *x*_1_ = *x*_4_ – *x*_7_; *x*_2_ = –*x*_4_ – *x*_7_; *x*_3_ = *x*_4_ – *x*_5_ – 2*x*_6_ + *x*_7_. By introducing four linearly independent vectors (1 0 0 0), (0 1 0 0), (0 0 1 0) and (0 0 0 –1) into (*x*_4_ *x*_5_ *x*_6_ *x*_7_), we obtain the four dimensionless numbers: Π_1_ = *Udρ/µ*, which is the Reynolds number (Re, inertia versus viscous force of the flow); Π_2_ = *l*/*d*, which is the length-to-diameter aspect ratio (i.e. slenderness) of the stem where deformations mainly occurred; Π_3_ = *A*/*d*^2^, which is the normalized area of the frond, and can be adjusted to the normalized projection area of the frond by Π_3_’ *= A*cos*θ*/*d*^2^, representing the capacity of the frondose part of the organism as a hydrodynamic load receiver and torque generator, regardless of potential tissue heterogeneity; and Π_4_ *= Uµ/dG*, which is the Ericksen number and, together with Π_1_, defines the maximum overall deflection or rotation of the stem under hydrodynamic loads derived mainly from the projected frondose area. It was found that the product of the latter three dimensionless numbers can predict the frond's turning angle, based on torsion theory. Their product is defined as the effective Ericksen number Er_eff_, which contains *J* (= *πd*^4^/32), the second polar moment of area, and *EJ* is the torsion rigidity (twice the flexural rigidity *EI*, where *I* is the second axial moment of area, *πd*^4^/64).

Therefore, we found that for frond–fluid interactions, the Reynolds number and the effective Ericksen number were the two key dimensionless numbers. To better quantify the flow, we used the frictional velocity to characterize this process. Thus, we have

(2.3) Ereff=μUτlAcos⁡(θ)(1+υ)16EJ=2μUτlAcos⁡(θ)(1+υ)Ed4;Reτ=Uτdρμ.

## 3. Results

### (a) Deformational modes

To characterize the deformation of *Charniodiscus* (*C*. *procerus* and *C*. *spinosus*) and thereby reconstruct its material properties, we examined 112 specimens from the Mistaken Point E surface [20], which included straight (figure 1A), bent (figure 1B) and torqued (figure 1C–F) forms (supplementary material, table S1), supplemented with specimens from the Mistaken Point North 2 surface to capture the preserved variation across surfaces. Among them, ‘kinked’ forms (i.e. a sudden change in the direction of central-line curvature; near the yellow arrows in figure 1C,D) were inferred to be typical of three-dimensional torsion (i.e. the organism was obliquely flipped over by 180° in three-dimensional space), rather than bending, based on the identification of a non-differentiable point at the connection between the frond and stem (see Methods) marked by a sudden change in curvature and the presence of two bucklings (i.e. unreleased tension; orange dashed box in figure 1C,D). These buckling features imply elastic behaviour and also allow us to rule out rheotactic growth during slow flow transitions as an explanation for the observed deformations: growth would have slowly released the tension, giving rise to a smooth frond-stem transition because non-differentiable points or buckling only occurs in growth under external mechanical or spatial constraints; for example, growth in rock cracks [57]. Moreover, naturally twisted morphogenesis under differential growth would produce either completely random twist directions, as seen in the plant *Vallisneria contortionis* or a fixed chirality with consistent wavelength. Neither of these patterns corresponds to what can be observed in *Charniodiscus*, which display consistent twist orientations and preferred falling directions, yet retain some variability likely arising from differences in the frond orientations during life relative to local flows. By contrast, fronds from other localities are generally undeformed, further indicating that the deformation observed at this site was a result of external mechanical forces rather than intrinsic growth.

The fronds had a predominantly solid stem (i.e. partly annular with a narrow hollow region) that extends into the frond with branches deriving monopodially from it, as supporting frameworks, rather than one with a thin wall (in contrast to the bag-like structure suggested for Charnia [10,13]). In thin-walled structures like those of Sabellidites, tunicates or even plastic drinking straws, structural integrity is extremely vulnerable to instability, leading to frequent inward buckling/deflation and angular kinks: localized deformations where the slender thin-walled structures abruptly bend into sharp-edged, often permanent, angles or folds with a collapsed/deflated surface (angular folds in ‘$/\overset{―}{X}\backslash$’ or ‘$\underset{―}{\overset{―}{X}}$’ shapes with deflation at the kinked/folded point; supplementary material, figure S2). However, across all mapped specimens [20], no known Charniodiscus fossils exhibit such abrupt angular folds along the main axis of the frond or deflated surfaces at inflexion points.

Based on the above understanding, we performed numerical simulations to explore how differing flow conditions and organismal material properties could give rise to the deformations observed in the fossils. Our initial analyses of the deformational modes demonstrated that the *Charniodiscus* with kinked forms were produced by the fronds flipping over in three-dimensional space during rapid burial, rather than forming from deformation of fronds already felled on the substrate (supplementary material, figure S3). Informed by observed specimen morphology, we carried out 73 simulations with different frictional velocities of the flow *U_τ_* (0.0005–0.015 m s^–1^), a reasonable range for the frictional velocity generated by contour currents [58], Young's modulus of the frond *E* (10–1000 kPa), angle between the initial normal vector of the frond face and the incoming flow *θ* (0–60°), and frond stem length *l* (0.124–0.152 m). In all these simulations, flow at the inlet transitioned from the frictional velocity *U_τ_* at the sediment-water interface into the linear and logarithmic regimes [47], with velocity approximately one order of magnitude higher than *U_τ_* at the mid-height of the frond. The frond was undeformed (i.e. straight) when fluid–structure interactions were weak (i.e. the flow was slow, the frond was short and/or the stalk diameter was large). In all other cases, the frond was deformed by flow, causing it to be knocked over onto the substrate. Three distinct modes of deformation were observed: bent (figure 2A), single torsion (‘kinked’ form, figure 2B), and secondary torsion (two smooth opposite curvatures along the stem, figure 2C), where *U_τ_* = 0.002 m s^–1^, *θ* = 0 and *E* = 100, 45 or 20 kPa, respectively. For fronds that underwent pure bending (figure 2A), the direction of curvature remained consistent from the stem to the frondose part, similar to multiple fossils on the Mistaken Point E surface (e.g. figure 1B), indicating that the elastic force within the stem was greater than the rotation of the frondose part caused by fluid drag and instability. However, for fronds that underwent single torsion (figure 2B), a mean ‘kink’ angle of approximately 137° between the frond and stem was observed across 21 simulations, comparable to the mean degree of torsion (approx. 134°) measured in 48 fossil specimens on the E surface (e.g. figure 1C,D). The initial sign of *θ* determined the frond's upstream side (e.g. the left side of the frond in figure 2D experienced higher flow velocity and thus pressure) and thus the torquing direction. High stress was concentrated at the stem–frond junction, with maximum stress at the stem–disk connection (figure 2E), indicating that the stem–disk junction would have been the most vulnerable to deformation and breakage, leading to commonly found isolated holdfasts [59]. Kinked forms always had two curvatures: a smooth one at the stem and an abrupt one at the stem–frond junction (figure 2B,E and figure 1C,D). These curvatures were accompanied by torsional bucklings, resulting in oblique (i.e. combined horizontal and vertical) displacements (figure 1C,D and figure 2B), as exemplified by the small buckling close to the stem–frond junction (orange dashed boxes in these figures).

**Figure 2.**
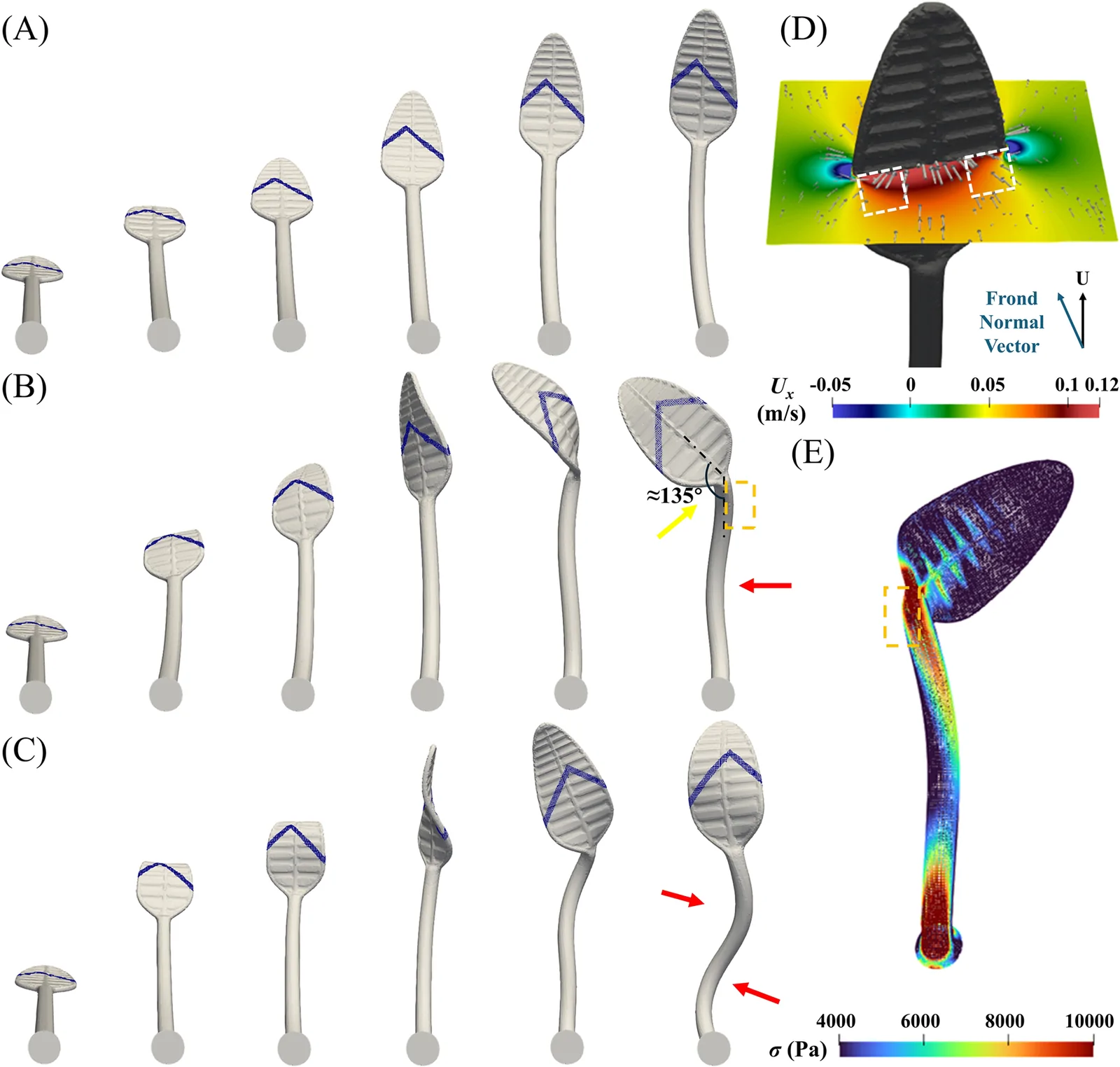
Numerical simulations of deformation in the Ediacaran frond *Charniodiscus*. (A–C) Upward views of deformed fronds that underwent (A) pure bending (elastic modulus, *E* = 100 kPa), (B) ‘kink’ under torsion (*E* = 45 kPa) and (C) S-shaped secondary torsion (*E* = 20 kPa). The blue arrows on the frond indicate the frond orientation, and the red and yellow arrows indicate the direction of curvature. The orange dashed boxes mark the small buckling close to the frond–stem connection, which is unique to unreleased torsion. The panels in each figure illustrate six successive stages of deformation before the frond was knocked down onto the substrate and reached a steady state. (D) Horizontal cross-section showing the *x*-component of flow velocity (*U_x_*) around a frond (θ = 15, *E* = 100 kPa, *U_τ_* = 0.0045 m s^–1^), with asymmetries in the flow velocity marked by the white boxes. (E) Downward view of a frond that underwent torsion (last stage in B) showing the von Mises stress field (*σ*) after falling.

If the frond was slender or flexible enough, a secondary torsion could occur after the first one had propagated downwards, causing two opposite smooth curvatures (S-shape) along the stem, also associated with buckling (figure 1E and figure 2C). In this case, the frond central line tended to be the extension of the second curvature. Such S-shaped or even U-shaped forms (under combined propagated torsion and transverse flow; see supplementary material, figure S4) were more common on the Mistaken Point North 2 surface (e.g. figure 1F), indicating that turbidity flows were stronger with a transverse component on this surface.

### (b) Scaling phase map and prediction of Young's modulus

To quantitatively characterize the main deformation modes and determine *Charniodiscus*’s material properties, we computed two dimensionless numbers using the Buckingham Π theorem [54]: the effective Ericksen number (Er_eff_), which relates to the angle of torsion, and the Reynolds number characterized by frictional velocity (Re*_τ_*) informing on the flow instability (see Methods). Using the results from the simulations, we created an Er_eff_*–*Re*_τ_* phase map (figure 3A) for the deformation modes: straight to bent (i.e. no torsion), temporary torsion (which would unfold), single torsion and secondary torsion.

**Figure 3.**
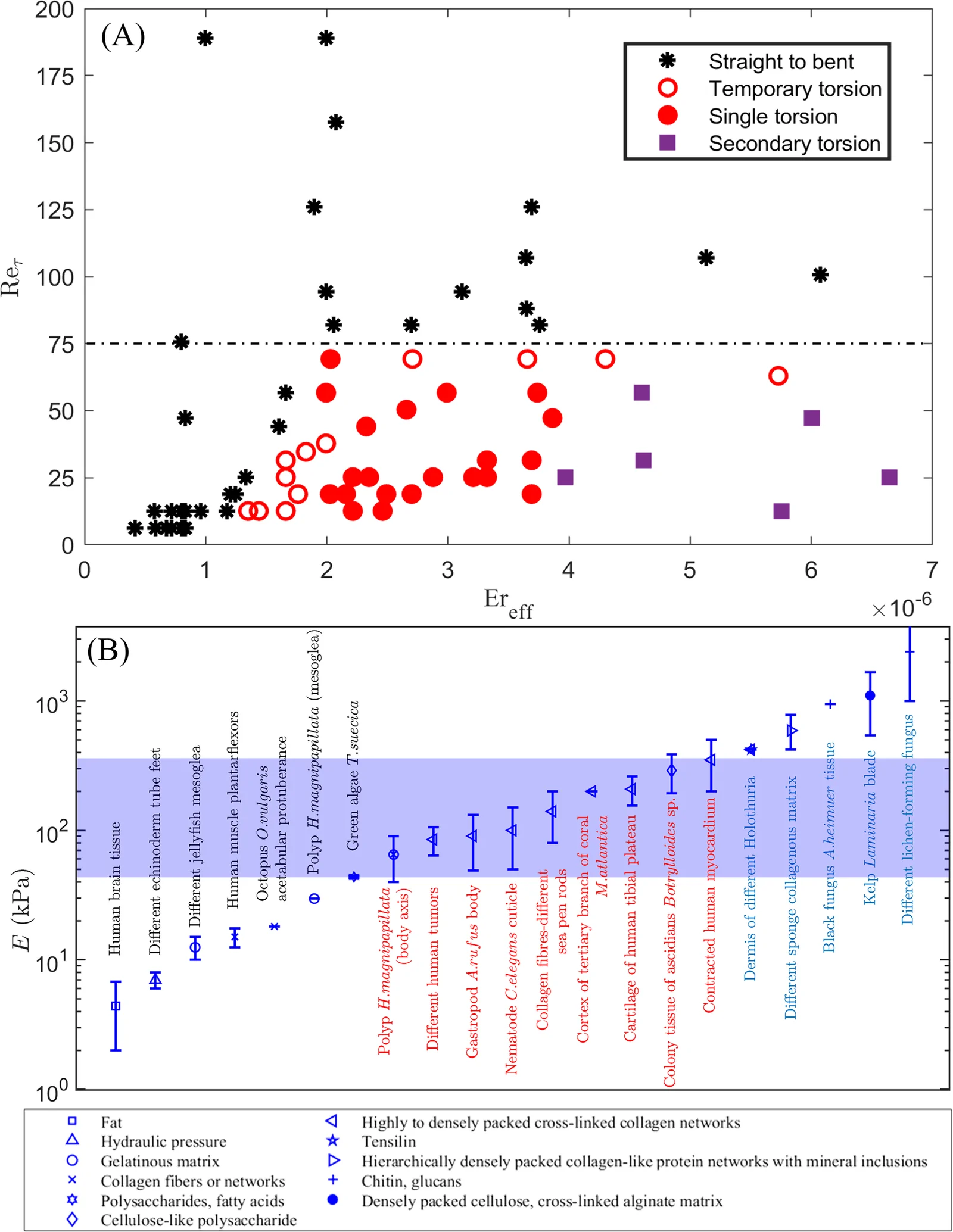
Identification of tissue material. (A) Phase map of the effective Ericksen number (Er_eff_) and the Reynolds number (Re*_τ_*) under different deformation modes of *Charniodiscus*: straight to bent, temporary torsion, single torsion and secondary torsion. (B) The Young's modulus (*E*) measured for extant eukaryotes compared with that estimated for *Charniodiscus* (blue shaded region), grouped by supporting material and/or structure/matrix. See table S2 in the supplementary material for full data. Taxa in red overlap with this estimated range.

As illustrated in the phase map (figure 3A), when Re*_τ_* > 75 (dashed line), torsion does not occur regardless of Er_eff_, and this is likely because there was insufficient time for torsion prior to the frond being knocked over. For Re*_τ_* < 65, the likelihood of torsion increases with Er_eff_ (i.e. an increase in stem length, frondose area, frictional/flow speed and/or a decrease in Young's modulus, stem diameter or deviation angle; see equation (2.3) in Methods). When the effective Ericksen number is approximately 1.5 × 10^–6^ < Er_eff_ < 2 × 10^–6^, some torsion could occur during falling, but this is not retained when the frond has been fully knocked over. When 2 × 10^–6^ < Er_eff_ < 4 × 10^–6^, some ‘kinked’ fronds are observed. When Er_eff_ > 2 × 10^–6^, S-shaped or even U-shaped secondary torsion can be seen. Since the frond size normally increases proportionally with the stem diameter [60] (Π*_3_*, see Methods), the length-to-diameter aspect ratio (*Π_2_*, see Methods) emerges as the key geometric factor for determining the deformational phase. *C*. *procerus* tends to have a slender stem, leading to more twisted forms (supplementary material, table S1), with extremely slender ones showing secondary torsion (e.g. figure 1E). *C*. *spinosus* tends to have a shorter and more robust stem that was more resistant to deformation under the same flow conditions, leaving most specimens preserved as straight (e.g. figure 1A) or slightly bent forms. This phenomenon strongly suggests the absence of active soft muscular movement and growth-induced curvature in the stem, which would have been independent of the stem profile and thus resulted in more diverse preserved forms even in short or stout specimens.

Based on our phase map, we can infer a range of Young's modulus values for *Charniodiscus*. Using the left frond in figure 1D and an inferred deep-water palaeoenvironment [21,22], typical flow rates are estimated at between 2 and 20 cm s^–1^ [58] (thus *U_τ_* > 0.00175m s^–1^). Torsion only occurred when Re*_τ_* < 65 (i.e. *U_τ_* < 0.008m s^–1^). Notably, 83.7% of the torqued fronds experienced dextral torsion. As frondose organisms are inferred to have exhibited rheotropism (i.e. growth predominantly perpendicular to the contour current to maximize the surface area of the frond exposed to flow) before they were knocked over, assuming the normal vectors of the fronds' surfaces follow a Gaussian distribution, only when the angle between the combined turbidity flow and contour currents and the mean orientation of the fronds' normal vector (parallel to the contour current direction owing to rheotropism) was –14.85° would 83.7% of the community have had a positive projection (–14.85° to approx. 45°) (see supplementary material, figure S5 for details). This result indicates that the flow that led to their death was a vectorized combination of the contour currents and turbidity flow. Incorporating all the measured geometrical parameters, estimated range of flow velocities and estimated mean angle of torsion into Er_eff_ gives a Young's modulus of 44.7–368.5 kPa.

## 4. Discussion

The studied *Charniodiscus* specimens underwent varying modes of deformation, including torsion, under different geometric and flow conditions. Our results indicate that Charniodiscus was not able to actively rotate the stem. By estimating Young's modulus (*E*) based on this deformation, we are able to make comparisons with the material properties of different eukaryotes, including lichen, fungi, algae, placozoans, cnidarians and tunicates [5–13], some of which have previously been compared with frondose organisms (figure 3B; supplementary material, table S2). Non-animal tissues such as those of kelp, fungi and lichen exhibit substantially higher Young's moduli (supplementary material, table S2), largely owing to the presence of cell walls composed of harder materials like cellulose or chitin [61]. The Young's modulus of the green alga *Tetraselmis suecia* falls within the lower bound of the range predicted for *Charniodiscus*; however, this is because they are unicellular organisms with cellulose; macroscopic organisms composed of the same material but lacking cell–cell linkage would be unable to withstand shear forces and would therefore be difficult to preserve intact under turbidites or even contour flows. Moreover, the deep-water environments inhabited by many *Charniodiscus* were below the photic zone, precluding photosynthesis as seen in extant photosynthetic micro and macroalgae [22,62], and this largely supports rejection of an algal affinity. A placozoan grade of organization [63] can also be rejected because in this phylum viscoelastic cell-cell adhesions [64,65] significantly influence overall tissue mechanics, making them prone to plasticity, ductility (being extremely out of shape) and brittleness (severely broken) under high tension (e.g. from water currents) [66]. This high plasticity is, again, inconsistent with the preserved morphology of *Charniodiscus*, which exhibits stable body outlines with no evidence of extensive distortion, collapse or breaking. Together, our results strongly support recent suggestions that *Charniodiscus* is a eumetazoan animal [67,68] to the exclusion of non-eumetazoan comparators [7,8].

Within eumetazoan animals, analogues for the material properties of *Charniodiscus* could be found within cnidarians. Among extant cnidarians, the mesoglea (a gelatinous extracellular matrix functioning as a hydrostatic skeleton) of scyphozoans and hydrozoans typically exhibits a Young's modulus of 10–30 kPa (supplementary material, table S2), which is lower than the range predicted for *Charniodiscus*. This softness results from their high-water content (>95%), low cell density and sparse collagen network, which enables neutral buoyancy and flexibility [26]. If *Charniodiscus* was composed solely of mesoglea, such softness would greatly impair its dynamic stability as an erect sessile organism, increasing the likelihood of preserved secondary torsion (e.g. if *E* = 20 kPa and *U_τ_* = 0.004 m s^–1^, then Er_eff_ = 2.11 × 10^–5^), but secondary torsion occurs in <3% of the observed specimens, all of which possess long and slender stems (e.g. figure 1E). However, the effective Young's modulus of the body axis of the hydrozoan *Hydra magnipapillata* (40–90 kPa; supplementary material, table S2) is greater than mesoglea *per se* and falls within the predicted range. This increased stiffness results from the presence of densely packed cells with well-developed cytoskeletal elements (actin and myosin filaments) in the epidermis and gastrodermis [69]. Similarly, the axial rod of the sea pen *Veretillum cynomorium* has a higher Young's modulus owing to the presence of closely packed, aligned collagen fibres, as well as calcite [70]. The stiffness of these dorsoventrally differentiated, symmetrical or glide-reflected colonial cnidarian structures enables them to maintain their posture in currents, in a manner similar to that inferred for *Charniodiscus*.

All the tissues within the predicted Young's modulus range for *Charniodiscus* (44.6–368.5 kPa) share a key component: densely packed cross-linked fibrous or meshed collagen. Parallel collagen fibre alignment optimizes load-bearing efficiency in one direction, while perpendicular and hierarchical arrangements strengthen the overall structure [71]. More condensed collagen networks give rise to enhanced stiffness, enabling the frond stem to have superior mechanical performance comparable to nematode cuticle, tumours and cartilage (supplementary material, table S2).

Given the preserved torsion in specimens, we infer that *Charniodiscus* could not have maintained an erect posture in flow if it was composed predominantly of mesoglea with high water content and sparse collagen, as in pelagic cnidarians and ctenophores (supplementary material, table S2). Instead, *Charniodiscus* must have had a dense, well-organized tissue structure. In modern animals, tissues with Young's moduli comparable to those estimated for *Charniodiscus* are typically composed of collagen fibres (supplementary material, table S2). Collagen synthesis is plesiomorphic for animals [72], with spongin—making up the mesohyl in sponges—consisting of a collagenous matrix made up of proteins similar and homologous to type IV collagens [73] (although we note that this is much harder (approx. 600 kPa) than the predicted range for *Charniodiscus* owing to the existence of cross-linking and mineralized spicules). However, it is only among eumetazoans that densely packed collagenous matrices are present [72], and the existence of these scaffolds underpins, at least in part, the evolution of tissue- and organ-grade anatomy [74], which allowed animals to diversify into myriad forms. Previous authors have suggested that some members of the frond-like Ediacaran macrobiota may represent at least tissue-grade animals [16] based on the presence of differentiated body structures, and our results confirm that some of the oldest Ediacaran fronds [75] already had the structural capacity for differentiated tissues.

In modern eumetazoans and homoscleromorph sponges, type IV collagens or homologous proteins are the primary supportive matrix to enhance structural stiffness [73]. Depending on the phylogenetic relationships among non-bilaterian lineages [76,77] and the interpretation of some fossil taxa (e.g. dinomischiids [78]), type IV collagen could represent the plesiomorphic condition of crown-group Eumetazoa, and densely packed collagenous matrices may have been acquired earlier along the stem lineage. The inferred load-bearing collagenous tissue of *Charniodiscus* is not compatible with what is known of sponge spongin, and the homoscleromorph mesohyl is a soft, jelly-like mesoglea. Likewise, ctenophores do not possess well-organized collagen networks, but rather amorphous extracellular matrices [79,80]. Therefore, as a non-living extracellular matrix, the load-bearing collagens in *Charniodiscus* stems were most likely secreted by an epidermal layer, as occurs in living animals such as octocorals (stems) [70]. Therefore, within a eumetazoan framework, these data support a minimally diploblastic-grade anatomy in *Charniodiscus*, but cannot distinguish between competing hypotheses of affinity within the Eumetazoa. This result is consistent with previous work suggesting the potential presence of collagenous tissues in other Ediacaran candidate eumetazoans, such as *Dickinsonia* [81] and *Pteridinium* [82].

The derived Young's modulus of these frondose organisms also provides critical insights into their dynamic interactions with ambient water flow. As demonstrated by the pioneering experimental study of *Charniodiscus* conducted by Singer *et al.* [83], models fabricated from a carrageenan–agar mixture—extracted polysaccharides (rather than native tissue) exhibiting a Young's modulus on the order of tens to a few hundred kPa [84,85] and comparable to our calculations—experienced substantially lower drag forces than their stiffer counterparts. At moderate-to-high flow velocities, such flexible structures would not only enable passive reorientation of the fronds towards the current direction, but also facilitate self-excited vibrations, which could have enhanced oxygen uptake and promoted material exchange at the frond surface [83]. Therefore, the derived Young's modulus corresponding to that of dense collagen fibres, rather than harder cellulose-based non-animal tissues, would allow these organisms to maintain a relatively erect posture under slow contour currents, while simultaneously reducing drag and improving nutrient uptake under relatively faster flow conditions. These mechanical advantages might have underpinned the ecological success and widespread prosperity of frondose communities during the terminal Ediacaran.

## 5. Conclusions

This study demonstrates how fossils of the frondose Ediacaran taxon *Charniodiscus* provide an opportunity to assess how torsion occurred during turbidity flow–organism interactions, establishing a novel methodological paradigm that infers tissue properties from structural deformation to illuminate the evolutionary origins and phylogenetic affinities of enigmatic early taxa. Through comparisons with extant eukaryotes, we can largely reject non-eumetazoan interpretations of *Charniodiscus* [5–13,63]. The tissues of *Charniodiscus*’s stem and frond main axis most closely resembled the densely aligned collagen fibres and particularly the axial rod of sea pens, without strongly hollow water-filled compartments or mesoglea, indicating a minimally diploblastic grade of anatomy. The presence of densely aligned collagen fibres in these early animals also marks a key evolutionary innovation, enabling the mechanical stability required for building larger and more complex structures, while at the same time facilitating dynamic but stable organism–environment interactions. The appearance of collagen fibres set the foundation for further evolutionary innovations.

## Supplementary Material



## Data Availability

Model set-ups and data are available at Zenodo [52]. Supplementary material is available online [86].

## References

[1] Liu AG , Kenchington CG, Mitchell EG. 2015 Remarkable insights into the paleoecology of the Avalonian Ediacaran macrobiota. Gondwana Res. 27, 1355–1380. (doi:10.1016/j.gr.2014.11.002)

[2] Boag TH , Darroch SAF, Laflamme M. 2016 Ediacaran distributions in space and time: testing assemblage concepts of earliest macroscopic body fossils. Paleobiology 42, 574–594. (doi:10.1017/pab.2016.20)

[3] Waggoner B . 2003 The Ediacaran Biotas in space and time. Integr. Comp. Biol. 43, 104–113. (doi:10.1093/icb/43.1.104) 21680415

[4] Mitchell EG , Pates S. 2025 From organisms to biodiversity: the ecology of the Ediacaran/cambrian transition. Paleobiology 51, 150–173. (doi:10.1017/pab.2024.21)

[5] Seilacher A . 1989 Vendozoa: organismic construction in the proterozoic biosphere. Lethaia 22, 229–239. (doi:10.1111/j.1502-3931.1989.tb01332.x)

[6] Retallack GJ . 1994 Were the Ediacaran fossils lichens? Palaeobiology 20, 523–544. (doi:10.1017/S0094837300012975)

[7] Peterson KJ . 2003 A fungal analog for Newfoundland Ediacaran fossils? Integr. Comp. Biol. 43, 127–136. (doi:10.1093/icb/43.1.127) 21680417

[8] Ford TD . 1958 Pre-Cambrian fossils from Charnwood Forest. Proc. Yorks. Geol. Soc. 31, 211–217. (doi:10.1144/pygs.31.3.211)

[9] Glaessner MF . 1959 Precambrian Coelenterata from Australia, Africa and England. Nature 183, 1472–1473. (doi:10.1038/1831472b0)

[10] Runnegar B . 2022 Following the logic behind biological interpretations of the Ediacaran biotas. Geol. Mag. 159, 1093–1117. (doi:10.1017/S0016756821000443)

[11] Dzik J . 2002 Possible ctenophoran affinities of the precambrian ‘Sea-Pen’ *Rangea.* J. Morphol. 252, 315–334. (doi:10.1002/jmor.1108) 11948678

[12] Richter R . 1955 Die ältesten Fossilien Südafrikas. Senckenb. Lethaea 36, 243–289.

[13] Butterfield NJ . 2022 Constructional and functional anatomy of Ediacaran rangeomorphs. Geol. Mag. 159, 1148–1159. (doi:10.1017/S0016756820000734)

[14] Runnegar B . 2022 Following the logic behind biological interpretations of the Ediacaran biotas. Geol. Mag. 159, 1093–1117. (doi:10.1017/S0016756821000443)

[15] Dunn FS , Liu AG, Gehling JG. 2019 Anatomical and ontogenetic reassessment of the Ediacaran frond *Arborea arborea* and its placement within total group Eumetazoa. Palaeontology 62, 851–865. (doi:10.1111/pala.12431)

[16] Dunn FS , Liu AG, Grazhdankin DV, Vixseboxse P, Flannery-Sutherland J, Green E, Harris S, Wilby PR, Donoghue PCJ. 2021 The developmental biology of *Charnia* and the eumetazoan affinity of the Ediacaran rangeomorphs. Sci. Adv. 7, eabe0291. (doi:10.1126/sciadv.abe0291) PMC8302126 34301594

[17] Evans SD , Droser ML, Erwin DH. 2021 Developmental processes in Ediacara macrofossils. Proc. Biol. Sci. 288, 20203055. (doi:10.1098/rspb.2020.3055) PMC7934905 33622124

[18] Cuthill JF , Morris SC. 2014 Fractal branching organizations of Ediacaran rangeomorph fronds reveal a lost proterozoic body plan. Proc. Natl Acad. Sci USA 111, 13122–13126. (doi:10.1073/pnas.1408542111) PMC4246981 25114255

[19] Bobrovskiy I , Krasnova A, Ivantsov A, Luzhnaya E, Brocks JJ. 2019 Simple sediment rheology explains the Ediacara biota preservation. Nat. Ecol. Evol. 3, 582–589. (doi:10.1038/s41559-019-0820-7) 30911145

[20] Stephenson NP , Delahooke KM, Barnes N, Rideout BWT, Kenchington CG, Manica A, Mitchell EG. 2024 Morphology shapes community dynamics in early animal ecosystems. Nat. Ecol. Evol. 8, 1238–1247. (doi:10.1038/s41559-024-02422-8) PMC11239517 38867093

[21] Benus AP . 1988Sedimentological context of a deep-water Ediacaran fauna (Mistaken Point, Avalon Zone, eastern Newfoundland). In Trace fossils, small shelley fossils and the Precambrian-Cambrian boundary, (eds E Landing, GM Narbonne, P Myrow), pp. 8–9. New York, NY: State Museum and Geological Survey Bulletin.

[22] Wood *et al.* 2003 Paleoenvironmental analysis of the late Neoproterozoic Mistaken Point and Trepassey formations, southeastern Newfoundland. Can. J. Earth Sci. 40, 1375–1391. (doi:10.1139/e03-048)

[23] Vixseboxse PB , Kenchington CG, Dunn FS, Mitchell EG. 2021 Orientations of mistaken point fronds indicate morphology impacted ability to survive turbulence. Front. Earth Sci. 9, 762824. (doi:10.3389/feart.2021.762824)

[24] Mitchell EG , Harris S, Kenchington CG, Vixseboxse P, Roberts L, Clark C, Dennis A, Liu AG, Wilby PR. 2019 The importance of neutral over niche processes in structuring Ediacaran early animal communities. Ecol. Lett. 22, 2028–2038. (doi:10.1111/ele.13383) PMC6899650 31515929

[25] Tramacere F , Kovalev A, Kleinteich T, Gorb SN, Mazzolai B. 2014 Structure and mechanical properties of Octopus vulgaris suckers. J. R. Soc. Interface 11, 20130816. (doi:10.1098/rsif.2013.0816) PMC3869157 24284894

[26] Veschgini M *et al.* 2023 Wnt/β-catenin signaling induces axial elasticity patterns of Hydra extracellular matrix. iScience 26, 106416. (doi:10.1016/j.isci.2023.106416) PMC10050647 37009232

[27] Novosel N *et al.* 2022 Salinity-induced chemical, mechanical, and behavioral changes in marine microalgae. J. Appl. Phycol. 34, 1293–1309. (doi:10.1007/s10811-022-02734-x)

[28] Wadamori Y , Vanhanen L, Savage GP. 2014 Effect of Kimchi fermentation on oxalate levels in silver beet (*Beta vulgaris* var. cicla). Foods 3, 269–278. (doi:10.3390/foods3020269) PMC5302357 28234318

[29] Denham JE , Ranner T, Cohen N. 2018 Signatures of proprioceptive control in *Caenorhabditis elegans* locomotion. Phil. Trans. R. Soc. 373, 20180208. (doi:10.1098/rstb.2018.0208) PMC6158217 30201846

[30] Essmann CL , Elmi M, Rekatsinas C, Chrysochoidis N, Shaw M, Pawar V, Srinivasan MA, Vavourakis V. 2024 The influence of internal pressure and neuromuscular agents on *C. elegans* biomechanics: an empirical and multi-compartmental *in silico* modelling study. Front. Bioeng. Biotechnol. 12, 1335788. (doi:10.3389/fbioe.2024.1335788) PMC10978802 38558792

[31] Bose S , Li S, Mele E, Silberschmidt VV. 2022 Exploring the mechanical properties and performance of type-I collagen at various length scales: a progress report. Materials 15, 2753. (doi:10.3390/ma15082753) PMC9025246 35454443

[32] Esford LE , Lewis JC. 1990 Stiffness of Caribbean gorgonians (Coelenterata, Octocorallia) and CaIMg content of their axes. Marine Ecol. Prog. Series 67, 189–200. (doi:10.3354/meps067189)

[33] Ma Y *et al.* 2024 Biomechanical properties of articular cartilage in different regions and sites of the knee joint: acquisition of osteochondral allografts. Cell Tiss. Bank. 25, 633–648. (doi:10.1007/s10561-024-10126-3) PMC11143059 38319426

[34] Zhang B *et al.* 2016 Biodegradable scaffold with built-in vasculature for organ-on-a-chip engineering and direct surgical anastomosis. Nat. Mater. 15, 669–678. (doi:10.1038/nmat4570) PMC4879054 26950595

[35] Motokawa T . 1984 Viscoelasticity of Holothurian body wall available to purchase. J. Exp. Biol. 109, 63–75. (doi:10.1242/jeb.109.1.63)

[36] Henry P . 2018 Variability and similarities in the structural properties of two related *Laminaria* kelp species. Estuar. Coast. Shelf. S. 200, 395–405. (doi:10.1016/j.ecss.2017.11.029)

[37] Monn MA , Kesari H. 2017 A new structure-property connection in the skeletal elements of the marine sponge *Tethya aurantia* that guards against buckling instability. Sci. Rep. 7, 39647. (doi:10.1038/srep39547) PMC5209657 28051108

[38] Wang S , Song W, Wang M, Wang J, Ding T, Zhou D, Ma S. 2022 Tests and analyses on physical and mechanical properties of fresh black fungus in picking season. PLoS ONE 17, e0275565. (doi:10.1371/journal.pone.0275565) PMC9560542 36227870

[39] Kochová P , Tonar Z. 2014 Structural and mechanical properties of gastropod connective and smooth muscle tissue. Exp. Mech. 54, 791–803. (doi:10.1007/s11340-014-9852-8)

[40] Mijailovic AS , Galarza S, Raayai-Ardakani S, Birch NP, Schiffman D, Crosby AJ, Cohen T, Peyton SR, Van Vliet KJ. 2021 Localized characterization of brain tissue mechanical properties by needle induced cavitation rheology and volume controlled cavity expansion. J. Mech. Behav. Biomed. 114, 104168. (doi:10.1016/j.jmbbm.2020.104168) 33218928

[41] Shinohara M , Sabra K, Gennisson J‐L, Fink M, Tanter M. 2010 Real-time visualization of muscle stiffness distribution with ultrasound shear wave imaging during muscle contraction. Muscle Nerve. 42, 438–441. (doi:10.1002/mus.21723) 20665510

[42] Fernández-Marín B *et al.* 2019 Symbiosis at its limits: ecophysiological consequences of lichenization in the genus Prasiola in Antarctica. Ann. Bot. 124, 1211–1226. (doi:10.1093/aob/mcz149) PMC6943718 31549137

[43] Woźnica A *et al.* 2022 The spatial structure (3D) and mechanical properties of the sponge *spongilla lacustris* L. (Porifera: Spongillida) skeleton as a potential tensegral architecture. Eur. Zool. J. 89, 1002–1017. (doi:10.1080/24750263.2022.2105964)

[44] Zhang J , Zhang T, Dong E, Zhang C, Lin Z, Song Z, Li H, Fang NX, Zhang Y. 2022 Bioinspired hydrogel jellyfish with mechanical flexibility and acoustic transparency. Cell Rep. Phys. Sci. 3, 101081. (doi:10.1016/j.xcrp.2022.101081)

[45] Cardiff P , Batistić I, Tuković Ž. 2025 solids4foam: a toolbox for performing solid mechanics and fluid-solid interaction simulations in OpenFOAM. JOSS 10, 7407. (doi:10.21105/joss.07407)

[46] Tuković Ž , Tuković Ž, Karač A, Cardiff P, Jasak H, Ivanković A. 2018 OpenFOAM finite volume solver for fluid-solid interaction. T. Famena 42, 1–31. (doi:10.21278/tof.42301)

[47] Schlichting H , Gersten K. 2000 Boundary-Layer theory, 8th edn. Berlin, Germany: Springer.

[48] Sharqawya MH , Lienhard JH, Zubair SM. 2010 Thermophysical properties of seawater: a review of existing correlations and data. Desalin. Water Treat. 16, 354–380. (doi:10.5004/dwt.2010.1079)

[49] Wilby P , Carney JN, Howe MPA. 2011 A rich Ediacaran assemblage from eastern Avalonia: evidence of early widespread diversity in the deep ocean. Geology 39, 655–658. (doi:10.1130/G31890.1)

[50] Nielsen JK , Bryan DR, Rand KM, Arostegui MC, Braun CD, Galuardi B, McDermott SF. 2023 Geolocation of a demersal fish (Pacific cod) in a high-latitude island chain (Aleutian Islands, Alaska). Anim. Biotelemetry 11, 29. (doi:10.1186/s40317-023-00340-3)

[51] Jourdain JS , Karač A, Cardiff P, Jasak H, Ivanković A. 2014 An image-based approach to extreme scale in situ visualization and analysis. In Proc. Int. Conf. for high performance computing, *networking, storage and analysis*, pp. 424–434. New Orleans, LA: IEEE Press.

[52] Wang Z , Buma-at P, Stephenson N, Dunn F, Zhuang Q, Mitchell E, Rahman I. 2026 Models and photos of Charniodiscus from Mistaken Point. Zenodo. (doi:10.5281/zenodo.17634882)

[53] Wang Z , Miguez-Salas O. 2025 Quantitative decoding of Ediacaran locomotory trace fossil morphologies: evidence for the emergence of slender anterior-posterior body profiles. Geology 292, 20250889. (doi:10.1130/G53332.1)

[54] Barenblatt GI . 2003 Scaling. Cambridge, UK: Cambridge University Press.

[55] Hang N , Wang Z, Liu M. 2023 A novel data-driven dimensional analysis framework for predicting melt pool morphology and porosity evolution in powder bed fusion. J. Mater. Process. Tech. 315, 117929. (doi:10.1016/j.jmatprotec.2023.117929)

[56] Zhang W , Soman P, Meggs K, Qu X, Chen S. 2013 Tuning the Poisson's ratio of biomaterials for investigating cellular response. Adv. Funct. Mater. 23, 3226–3232. (doi:10.1002/adfm.201202666) PMC3785557 24076754

[57] Whiteley GM , Hewitt JS, Dexter AR. 1982 The buckling of plant roots. Physiol. Plant. 54, 333–342. (doi:10.1111/j.1399-3054.1982.tb00268.x)

[58] Heezen BC , Hollister CD, Ruddiman WF. 1966 Shaping of the continental rise by deep geostrophic contour currents. Science 152, 502–508. (doi:10.1126/science.152.3721.502) 17815077

[59] Burzynski G , Narbonne GM, Alexander Dececchi T, Dalrymple RW. 2017 The ins and outs of Ediacaran discs. Precam. Res. 300, 246–260. (doi:10.1016/j.precamres.2017.08.012)

[60] LaFlamme M , Narbonne GM, Anderson MM. 2004 Morphometric analysis of the Ediacaran frond *Charniodiscus* from the Mistaken Point Formation, Newfoundland. J. Paleont. 78, 827–837. (doi:10.1666/0022-3360(2004)078<0827:MAOTEF>2.0.CO;2)

[61] Samadam A , Vallepalli R, Neeraj Kumar K, Sreekanth M, Goud R Raman. 2022 Mechanical properties evaluation and behaviour of cellulose-HDPE composite. Mater. Today Proc. 62, 3405–3410. (doi:10.1016/j.matpr.2022.04.271)

[62] Falkowski PG , Raven JA. 2007 Aquatic photosynthesis, 2nd edn. Princeton, NJ: Princeton University Press.

[63] Dufour SC , McIlroy D. 2016 Ediacaran pre-placozoan diploblasts in the Avalonian biota: the role of chemosynthesis in the evolution of early animal life. Geol. Soc. Spec. Publ. 448, 211–219. (doi:10.1144/SP448.5)

[64] Lenne P , Rupprecht J, Viasnoff V. 2021 Cell junction mechanics beyond the bounds of adhesion and tension. Dev. Cell 56, 202–212. (doi:10.1016/j.devcel.2020.12.018) 33453154

[65] Smith CL , Reese TS, Govezensky T, Barrio RA. 2019 Coherent directed movement toward food modeled in Trichoplax, a ciliated animal lacking a nervous system. Proc. Natl Acad. Sci USA 116, 8901–8908. (doi:10.1073/pnas.1815655116) PMC6500112 30979806

[66] Prakash VN , Bull MS, Prakash M. 2021 Motility-induced fracture reveals a ductile-to-brittle crossover in a simple animal's epithelia. Nat. Phys. 17, 504–511. (doi:10.1038/s41567-020-01134-7)

[67] Park T-Y , Woo J, Lee D-J, Lee D-C, Lee S-B, Han Z, Chough SK, Choi DK. 2011 A stem-group cnidarian described from the mid-cambrian of China and its significance for cnidarian evolution. Nat. Commu. 2, 440. (doi:10.1038/ncomms1457) PMC3265377 21863009

[68] Budd GE , Jensen S. 2017 The origin of the animals and a ‘Savannah’ hypothesis for early bilaterian evolution. Biol. Rev. 92, 446–473. (doi:10.1111/brv.12239) 26588818

[69] Leclère L , Röttinger E. 2017 Diversity of cnidarian muscles: function, anatomy, development and regeneration. Front. Cell Dev. Biol. 4, 157. (doi:10.3389/fcell.2016.00157) PMC5253434 28168188

[70] Ledger PW , Franc S. 1978 Calcification of the collagenous axial skeleton of *Veretillum cynomorium* Pall. (Cnidaria: Pennatulacea). Cell Tiss. Res. 192, 249–266. (doi:10.1007/BF00220743) 29708

[71] Puetzer JL , Ma T, Sallent I, Gelmi A, Stevens MM. 2021 Driving hierarchical collagen fiber formation for functional tendon, ligament, and meniscus replacement. Biomaterials 269, 120527. (doi:10.1016/j.biomaterials.2020.120527) PMC7883218 33246739

[72] Fidler AL *et al.* 2017 Collagen IV and basement membrane at the evolutionary dawn of metazoan tissues. eLife 6, e24176. (doi:10.7554/eLife.24176) PMC5395295 28418331

[73] Aouacheria A , Geourjon C, Aghajari N, Navratil V, Deleage G, Lethias C, Exposito J-Y. 2006 Insights into early extracellular matrix evolution: spongin short chain collagen-related proteins are homologous to basement membrane type IV collagens and form a novel Family widely distributed in invertebrates. Mol. Biol. Evol. 23, 2288–2302. (doi:10.1093/molbev/msl100) 16945979

[74] Hynes RO . 2012 The evolution of metazoan extracellular matrix. J. Cell Biol. 196, 671–679. (doi:10.1083/jcb.201109041) PMC3308698 22431747

[75] Matthews JJ , Liu AG, Yang C, McIlroy D, Levell B, Condon DJ. 2021 A chronostratigraphic framework for the rise of the Ediacaran macrobiota: new constraints from Mistaken Point Ecological Reserve, Newfoundland. Geol. Soc. Am. Bull. 133, 612–624. (doi:10.1130/B35646.1)

[76] Pisani D , Pett W, Dohrmann M, Feuda R, Rota-Stabelli O, Philippe H, Lartillot N, Wörheide G. 2015 Genomic data do not support comb jellies as the sister group to all other animals. Proc. Natl Acad. Sci USA 112, 15402–15407. (doi:10.1073/pnas.1518127112) PMC4687580 26621703

[77] Whelan NV , Kocot KM, Moroz LL, Halanych KM. 2015 Error, signal, and the placement of Ctenophora sister to all other animals. Proc. Natl Acad. Sci USA 112, 5773–5778. (doi:10.1073/pnas.1503453112) PMC4426464 25902535

[78] Zhao Y , Vinther J, Parry LA, Wei F, Green E, Pisani D, Hou X, Edgecombe GD, Cong P. 2019 Cambrian sessile, suspension feeding stem-group ctenophores and evolution of the comb jelly body plan. Current Biol. 29, 1112–1125.e2. (doi:10.1016/j.cub.2019.02.036) 30905603

[79] Hernandez-Nicaise M , Nicaise G, Reese TS. 1989Intercellular junctions in ctenophore integument. In Evolution of the first nervous systems, (ed. PAV Anderson), Boston, MA: Springer.

[80] Chioran A , Duncan S, Catalano A, Brown TJ, Ringuette MJ. 2017 Collagen IV trafficking: the inside-out and beyond story. Dev. Biol. 431, 124–133. (doi:10.1016/j.ydbio.2017.09.037) 28982537

[81] Evans SD , Huang W, Gehling JG, Kisailus D, Droser ML. 2019 Stretched, mangled, and torn: responses of the Ediacaran fossil *Dickinsonia* to variable forces. Geology 47, 1049–1053. (doi:10.1130/G46574.1)

[82] Meyer M *et al.* 2014 Three-dimensional microCT analysis of the Ediacara fossil *Pteridinium simplex* sheds new light on its ecology and phylogenetic affinity. Precambrian Res. 249, 79–87. (doi:10.1016/j.precamres.2014.04.013)

[83] Singer A , Plotnick R, Laflamme M. 2012 Experimental fluid mechanics of an Ediacaran frond. Palaeontol. Electron. 15, 1–5. (doi:10.26879/297)

[84] Fujita H , Ninomiya K, Homma T. 1952 Mechanical properties of concentrated hydrogels of agar-agar. I. Modulus of elasticity in compression. Bull. Chem. Soc. Jpn. 25, 374–378. (doi:10.1246/bcsj.25.374)

[85] Kozbial A , Li L. 2017 Study on the friction of κ-carrageenan hydrogels in air and aqueous environments. Mater. Sci. Eng. C 36, 173–179. (doi:10.1016/j.msec.2013.12.003) 24433901

[86] Wang Z , Buma-at P, Stephenson N, Dunn FS, Zhuang Q, Mitchell EG, Rahman IA. 2026 Supplementary material from: Biomechanical analyses point to dense collagen fibre architecture in an Ediacaran frond. Figshare. (doi:10.6084/m9.figshare.c.8694628) PMC13678724 42869480

