# Supplementary material for: Biomechanical analyses point to dense collagen fibre architecture in an Ediacaran frond
Source: Proc Biol Sci. 2026 Oct 14;293(2080):20261630. doi: 10.1098/rspb.2026.1630 (PMC13678724; doi:10.1098/rspb.2026.1630)
Supplement: Supplementary file 1 [file rspb.2026.1630.zip › rspb.2026.1630/rspb20261630_si_001.docx]

**Supplement for**

**“Biomechanical analyses point to dense collagen fibre architecture in an Ediacaran frond”**

Zekun Wang^1*^, Princess Buma-at^2^, Nile Stephenson^2,3^, Frances S. Dunn^4^, Qianze Zhuang^5^, Emily G. Mitchell^2^, Imran A. Rahman^1,4^

^1^ The Natural History Museum, London SW7 5BD, UK

^2^ Department of Zoology and University Museum of Zoology, University of Cambridge, Cambridge CB2 3EJ, UK

^3^ King’s College, University of Cambridge, Cambridge, CB2 1ST, UK

^4^ Oxford University Museum of Natural History, University of Oxford, Oxford OX1 3PW, UK

^5^ State Key Laboratory of Coastal and Offshore Engineering, Dalian University of Technology, Dalian 116024, China

^*^ Corresponding author: zekun.wang@nhm.ac.uk

**Supplementary Figures and Tables**

**Fig. S1.**

**Verification of the solver.** **(A)** Flowchart summarizing the computational process. **(B, C)** Benchmark case. Deformation of a pure round beam (with a diameter matching that of the frond model) in a uniform current (*U* = 0.04 m/s) (**C**) compared with the analytical solution of the deflection **(B)**. In the benchmark, the Young’s Modulus (*E*) of the beam is 10000 Pa, beam length is 0.125 m, density (*ρ*) is 1000 kg/m^3^ and viscosity (*μ*) is 0.001 kg/(m∙s). At a Reynolds number of 500, the drag coefficient of a cylinder (*C*_d_) is ~1.2. The uniform load (*q*) that the current exerts on the beam is: $q=\frac{1}{2l}C_{\text{d}}A\rho U^{2}=\frac{1}{2l}C_{\text{d}}dl\rho U^{2}=0.6\rho dU^{2},$ where *A* is the projection area of the beam (*dl*). Solving the Euler–Bernoulli equation gives the deflection curve of the beam as: $x=\frac{qh^{2}}{24EI}\left(h^{2}+6l^{2}-4hl\right)=41.7215h^{2}(h^{2}-0.5h+0.09375)$, where *x* is the lateral displacement and *h* is the height above the ground. The numerical results align well with the analytical solution, verifying the solver and providing a solid foundation for our subsequent numerical studies. **(D)** The sensitivity test on the results, where deformation barely changed when simulation domain doubles.

**Fig. S2.**

**Twisted *Sabellidites* and rubber tubes.**

(A) *Sabellidites* with ‘’ or ‘’ -shaped kinks. The black arrows indicate kinks/folds and the yellow dashed line indicates the flipping axis. (B) Deflated (inward buckling) of a rubber tube. (C) ‘’-shaped kinks in a flipped rubber tube with angular edges caused by deflation. These phenomena are evident despite silicone rubber having a higher Young’s modulus; a softer tube would experience more severe deflation and angular kinks. Scale bars: 1 cm.

**Fig. S3.**

**Deformations of fronds with recumbent postures and related discussions.**

We performed simulations and found that oblique flow, containing components parallel or perpendicular to the stem, acting on a recumbent frondose organism, whether fallen after death (or living with a flexible recumbent posture [85]), produces bending **(A)** and/or coaxial axial rotation **(B)**. These outcomes differ from the observed kinked forms that contain a non-differentiable point (abrupt change in orientation). **(A)** Bending occurs when the frond can resist the flow to some degree, such as in relatively slow currents or when the frond possesses higher stiffness; **(B)** Co-axial rotation occurs when the frond offers little resistance to uniform flow. This co-axial rotation translates into rolling due to substrate friction. Because the frond and stem share the same Principal Axis of Inertia, the rolling axis of the frond coincides with the axis of the stem. As a result, the recumbent structure remains relatively straight at distal part while rolling around the holdfast, producing an arc or circular scratch mark when the substrate permits [86]. Rolling leads to the accumulation of rotational displacement and eventually to material fatigue, causing breakage at the stem–disk connection. Such failure would be fatal for a living organism in a recumbent posture, but it is consistent with post-mortem deformation of a fallen frondose organism. These results, together with the simulations of erect fronds presented below in the manuscript, indicate that the observed kinked fronds require three-dimensional torsion rather than deformation upon a horizontal substrate.

**Fig. S4.**

**Numerical simulation of a frond that underwent U-to-S-shaped deformation.** When *U_τ_* = 0.0038 m/s, *θ* = 15°, and *E* = 25 kPa, the torsion propagated downwards accompanied by lateral deformation due to the inclusion of a transverse flow component. The blue arrows on the frond indicate the frond orientation.

**Fig. S5.**

**Flow direction controls frond torsional direction from their default orientation.** *Charniodiscus* is inferred to exhibit rheotropism, with the normal vectors of the fronds approximately parallel to the daily flow direction they experienced (i.e. contour currents) under a Gaussian-distributed orientation, which would have served to maximize the surface area exposed to flow for feeding or respiration. If the frond default orientation is <–45° or >45, there would be no benefit from exposure to the contour flow due to the projection area being smaller than the side area. Therefore, it is assumed that the natural frond orientation under normal flow conditions (i.e. contour currents) was statistically limited to within ±45°, with a three-sigma confidence interval. In this case, only when the orientation of the turbidity flow was 14.85° left to the mean normal vector of the frond would 83.7% of the fronds encounter flow from the right-backside first, leading to the anticlockwise torsion observed in our simulations.

**Table S1.**

|  | Straight | Bent | Single torsion | Secondary torsion |
| --- | --- | --- | --- | --- |
| *C*. *procerus* | 8 | 17 | 44 | 3 |
| *C*. *spinosus* | 26 | 9 | 5 | 0 |

**Number of occurrences of different deformational modes of *Charniodiscus* on the Mistaken Point E surface.**

**Table S2.**

| Tissue | | Effective Young’s modulus (kPa) | | Main material and/or supportive structure | | References | |
| --- | --- | --- | --- | --- | --- | --- | --- |
| Brain tissue | | 3–5.8 | | ~60% weight is fat, rest mainly water, protein, carbohydrates | | Mijailovic et al. 2021 | |
| Echinoderm tube feet | | 6–8 | | Fluid-filled hydrostatic structure | | Tramacere et al. 2014 | |
| Jellyfish mesoglea | | 10–15 | | Gelatinous matrix, very sparce collagens | | Tramacere et al. 2014; Zhang et al., 2022 | |
| Human muscle | | 12.5–17.5 | | Unidirectional Type I collagen fibers | | Shinohara et al. 2020 | |
| Octopus acetabular protuberance | | 18.1 | | Type I collagen network | | Tramacere et al. 2014 | |
| *H. magnipapillata* (mesoglea) | | 29.7 | | Gelatinous matrix, collagen networks | | Veschgini et al. 2017 | |
| Green algae *T. suecica* | | 42.8–44.7 | | Polysaccharides, fatty acids | | Novosel et al. 2022 | |
| *H. magnipapillata* (body axis) | | 40–90 | | Epidermis with contractile Type I collagen networks, gelatinous mesoglea | | Veschgini et al. 2017 | |
| Different tumors | | 64–106 | | Highly cross-linked Type I, III collagen network | | Wadamori 2014 | |
| Gastropod body axis (large deformations) | | 49–132 | | Highly cross-linked Type I collagen network | | Kochová & Tonar 2014 | |
| Nematode *C. elegans* cuticle | | 50–150 | | Densely cross-linked, collagen-like proteins and non-collagenous cuticulins | | Denham et al. 2018, Essmann et al. 2023 | |
| Collagen fibres (e.g. sea pen rods) | | 80–200 | | Highly aligned cross-linked, collagen fibers | | Franc et al. 1985; Bose et al. 2022 | |
| Cortex of tertiary branch of coral M. atlantica | | 200 | | Highly aligned cross-linked, collagen fibers with certain mineral contents | | Esford and Lewis 1990 | |
| Cartilage of tibial plateau | 155.73–260.91 | | | | Dense, three-dimensional Type II collagen networks | Ma et al. 2024 | |
| Ascidians *Botrylloides*: | | 194–386 | | Cellulose-like polysaccharide | | Edlund and Koehl 1998 | |
| Myocardium (contracted) | | 200–500 | Contracted tightly packed cross-linked collagen fibers networks | | | | Zhang et al. 2016 |
| Dermis of *Holothuria* | | 420 | | Densely packed, cross-linkable diverse types of collagen networks with tensilin dynamically bridging collagen fibrils | | Motokawa 1984 | |
| Sponge collagenous matrix (spongin) | | 590 (SD = 180) | | Densely and hierarchically packed collagen-like protein network, often with mineral inclusions | | Monn and Kesari 2017; Woźnica et al., 2022 | |
| Black fungus | | 947 | | Chitin, glucans | | Wang et al. 2022 | |
| Kelp blade | | 540–1660 | | Densely packed cellulose, cross-linked alginate matrix | | Henry 2018 | |
| Lichen | 1000–3800 | | | Chitin, glucans, cellulose | | Fernández-Marín 2019 | |

**Young’s modulus for different biological tissues.**
